# Grouping based feature attribution in metacontrast masking

**DOI:** 10.2478/v10053-008-0018-z

**Published:** 2008-07-15

**Authors:** Thomas U. Otto

**Affiliations:** Laboratory of Psychophysics, Brain Mind Institute, Ecole Polytechnique Fédérale de Lausanne (EPFL), Switzerland

**Keywords:** metacontrast masking, feature attribution, feature integration, motion grouping, attention

## Abstract

The visibility of a target can be strongly suppressed by metacontrast masking. Still, some features of the target can be perceived within the mask. Usually, these rare cases of feature mis-localizations are assumed to reflect errors of the visual system. To the contrary, I will show that feature "mis-localizations" in metacontrast masking follow rules of motion grouping and, hence, should be viewed as part of a systematic feature attribution process.

## Feature mis-localization in metacontrast masking

**Figure 1.**
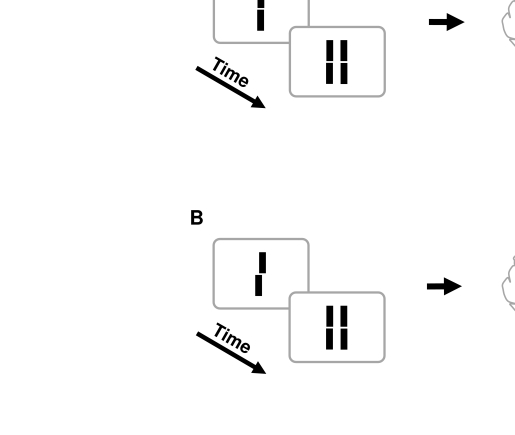
Classical metacontrast masking. (A) A central line is followed by two, non-overlapping flanking lines. The central line is rendered largely invisible if the flanks appear about 50 ms later. (B) Feature mis-localization in metacontrast masking. Similar to A, a central offset line is followed by two aligned flanks. Although the visibility of the central line is strongly suppressed, its offset is bequeathed to the flanking lines. Adapted from Otto et al. (2006) with permission, ©ARVO.

In metacontrast masking, the visibility of a target is reduced by a temporally succeeding and spatially non-overlapping mask (Alpern, 1953; Stigler, 1910; for a recent monograph see Breitmeyer & Öğmen, 2006). Metacontrast masking yields non-monotonic U-shaped masking functions, that is, performance on the target is most deteriorated for intermediate SOAs. For example, if a single line is followed by a pair of flanking lines after about 50 ms, only the flanking lines are perceived (Figure 1A). However, the target is clearly visible if the SOA is either very short (e.g. 0 ms) or very long (>150 ms).

**Figure 2.**
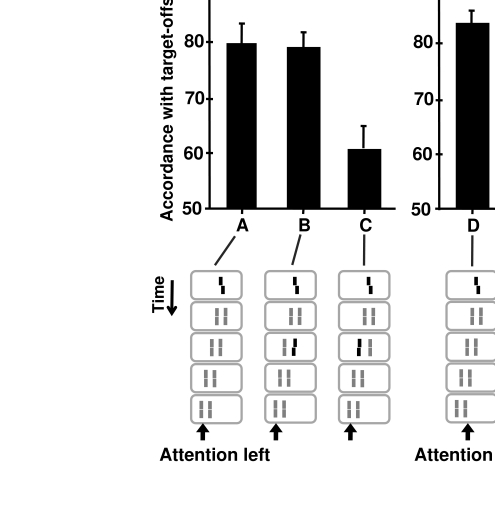
Sequential metacontrast. The central target line is followed by a sequence of flanking lines, here by two streams of lines shifting to the left. (A) Observers were asked to report the offset of the attended left stream of lines. If only the target line is randomly offset to the left or right, a corresponding offset direction is reported pre-dominantly. (B, C) A second offset in the opposite direction is presented either at the right (B) or left line (C) in the third frame. Performance, compared to A, is changed if the second offset is presented to the left line (C). Performance is not changed in B although the second offset is presented at the same spatial position as the target. (D-F) Stimuli are exactly the same as in A-C, respectively. Observers were asked to attend to the right stream of lines. Similar to A, if only the target line is offset, a corresponding offset is reported (D). However, feature integration in E and F is reversed compared to B and C. Performance compared to D is changed by the offset presented at the right line (E), whereas performance is only slightly changed by the second offset presented at the left line (F). These findings indicate that a small leakage across motion steams is possible. Still, features are basically integrated within the attended motion streams. A, B, D, and E adapted from Otto et al. (2006) with permission, ©ARVO.

Surprisingly, when the target line itself is invisible, some features of the suppressed target can be perceived as mis-localized within the flanking lines (Figure 2B). Werner (1935) was the first to observe feature mis-localizations in metacontrast masking. When he presented a polygon followed by a surrounding ring, the ring appeared as a “ring with teeth” (Werner, 1935, p. 58). Similarly, there are other anecdotal reports of feature mis-localization (e.g., Hogben & Di Lollo, 1984; Stewart & Purcell, 1970; Stoper & Banffy, 1977), but only a few systematic studies (Hofer, Walder, & Groner, 1989; Wilson & Johnson, 1985). It has been shown that not only contour features of a target can be inherited but also brightness (Burr, 1984; Toch, 1956), and that the duration of an invisible target can contribute to the perceived duration of the following mask (Scharlau, this volume). Moreover, feature mis-localizations can occur in pattern masking (Herzog & Koch, 2001). In summary, although the visibility of a target can be strongly reduced in metacontrast masking, several features of the target can be perceived within the mask. Here, the question arises, if the target itself is suppressed, how are these features processed?

## Feature attribution is determined by motion grouping

Recently, Otto, Öğmen, and Herzog (2006) introduced a paradigm, coined *sequential metacontrast*, to study feature attribution in metacontrast masking. In sequential metacontrast, a target line is not only followed by one pair of flanking lines (as in Figure 1A), but by sequences of lines. These sequences elicit the percept of lines in apparent motion, whereas the target line is not visible itself. If the first line is offset (as in Figure 1B), this offset can be perceived to be mis-localized in the motion streams in a rather broad spatial window up to

0.5 deg (Otto et al., 2006). Interestingly, if multiple offsets are presented within one stream of lines, these offsets can be integrated with the target line offset. Importantly, this feature integration occurs only within a grouped motion stream. For example in Figure 2, two streams of lines shifting in the same direction are presented after the display of the target line. If the target line is offset, observers report a corresponding offset in both the left and the right motion stream (Figure 2A, D). However, if a second offset is added non-ambiguously either to the right (Figure 2B, E) or left motion stream (Figure 2C, F), performance strongly differs depending on which stream was attended, although the physical stimulus is exactly the same. Hence, two offsets, even if they are presented at the same spatial location as in Figure 2B, E, are only integrated if they belong to the same attended motion stream.

To summarize, the visibility of a target can be strongly suppressed by sequential metacontrast masking. However, although the target line itself is invisible, its offset can be perceived as mis-localized within the masking lines. Usually, the rare cases of feature mis-localizations are interpreted to reflect limitations or errors of the visual system. For example, illusory conjunctions- the incorrect perceptual combinations of correctly perceived features like color and shape- usually occur when the observer’s attention is diverted (Treisman & Schmidt, 1982). Consequently, this illusory feature mis-localization has been interpreted to result from limited attentional resources. Similarly, feature mis-localizations in metacontrast masking might be explained in terms of a limited processing capacity of the visual system unable to cope with the fast rate of stimulus presentations. However, the selectivity of feature integration in sequential metacontrast indicates that grouping operations can access and process individual features prior to an integration stage (Figure 2). Hence, the feature “mis-localizations” in sequential metacontrast masking should not be viewed as errors of the visual system, but rather as part of a systematic process of feature attribution determined by attention and motion grouping (Öğmen, Otto, & Herzog, 2006; Otto et al., 2006). The exact underlying mechanisms- while possibly involving recurrent processing as proposed by Hamker (this volume) – have to be unearthed in future research.
